# Structure of *N*-acetylglucosamine-1-phosphate uridyltransferase (GlmU) from *Mycobacterium tuberculosis* in a cubic space group

**DOI:** 10.1107/S1744309109010252

**Published:** 2009-04-24

**Authors:** Sunil Kumar Verma, Mamta Jaiswal, Neeraj Kumar, Amit Parikh, Vinay Kumar Nandicoori, Balaji Prakash

**Affiliations:** a Department of Biological Sciences and Bioengineering, Indian Institute of Technology, Kanpur 208016, India; b National Institute of Immunology, Aruna Asaf Ali Marg, New Delhi 110067, India

**Keywords:** *Mycobacterium tuberculosis*, bifunctional enzymes, acetyltransferases, uridyltransferases, GlmU

## Abstract

GlmU is a bifunctional enzyme that catalyzes the final two steps in the biosynthesis of UDP-GlcNAc. Crystals of GlmU from *Mycobacterium tuberculosis* obtained using ammonium sulfate as a precipitant diffracted poorly (to 3.4 Å resolution) and displayed an unusually high solvent content (>80%) with sparse crystal packing that resulted in large solvent channels. With one molecule per asymmetric unit, the monomers from three neighbouring asymmetric units related by the crystal threefold formed a biological trimer. Although this is the first report of the structure of GlmU determined in a cubic crystal form, the trimeric arrangement here is similar to that observed for other GlmU structures determined in hexagonal (*H*3, *H*32, *P*6_3_22) space groups.

## Abstract 2

The structure of *M. tuberculosis* *N*-acetylglucosamine-1-phosphate uridyltransferase (GlmU) was determined by the molecular-replacement method to 3.4 Å resolution in space group *I*432 and was refined to a final *R*_work_ and *R*_free_ of 0.285 and 0.321, respectively.

## 1. Introduction

The *Rv1018c* (*glmU*) gene product of *Mycobacterium tuberculosis* is an *N*-acetylglucosamine-l-phosphate uridyltransferase (GlmU). GlmU, a bifunctional enzyme, catalyzes the final two steps (reactions 1 and 2 below) in the *de novo* biosynthesis of UDP-GlcNAc from acetyl-CoA, glucosamine-1-phosphate and UTP,
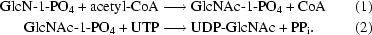
While the N-terminal domain of GlmU catalyzes uridyltransferase activity (reaction 2 above), acetyltransferase activity (reaction 1 above) at the C-terminal domain requires the formation of a bio­logical trimer.

UDP-GlcNAc is an essential precursor for the biosynthesis of peptidoglycan and lipopolysaccharide, which are constituents of the bacterial cell wall (Barreteau *et al.*, 2008 ▶; Zhang *et al.*, 2008 ▶). With the emergence of mycobacterial multiple drug resistance during the treatment of tuberculosis, the biosynthetic pathway of UDP-GlcNAc might present an alternative target for new antibacterial agents. Recently, we reported modulation of the acetyltransferase activity of *M. tuberculosis* GlmU upon phosphorylation by the eukaryotic-like serine-threonine protein kinase B (PknB; Parikh *et al.*, 2008 ▶).

The crystal structures of GlmU from *Escherichia coli*, *Streptococcus pneumoniae*, *Haemophilus influenzae* and *M. tuberculosis* have previously been reported in the hexagonal space groups *H*3, *H*32 and/or *P*6_3_22 to resolutions better than 2.8 Å (Olsen & Roderick, 2001 ▶; Sulzenbacher *et al.*, 2001 ▶; Mochalkin *et al.*, 2008 ▶). Here, we report that GlmU also crystallizes in the cubic space group *I*432. However, it only diffracts to 3.4 Å resolution. This poor diffraction is correlated with a sparse crystal packing leading to the presence of large solvent channels in the crystal, unlike the hexagonal forms. The distinct crystal packing in these two forms seems to be a result of the involvement of different surfaces in crystal contacts.

## 2. Material and methods

### 2.1. Cloning, expression and purification

The gene encoding GlmU (*Rv1018c*, *glmU*) was cloned into a pQE2 (Qiagen) expression vector and the protein was purified as described in Parikh *et al.* (2008 ▶). Briefly, plasmid pQE2-GlmU was transformed in *E. coli* DH5α and the cells were grown in Luria broth (with 100 µg ml^−1^ ampicillin) and induced with 0.1 m*M* IPTG. Cells were lysed by sonication in phosphate buffer containing 5% glycerol, 1 m*M* β-mercaptoethanol and HIS-cocktail (Sigma). Clarified cell lysate was loaded onto a pre-equilibrated Ni–NTA column (His-Trap FF GE Healthcare). The protein was eluted using a linear gradient of imidazole to 500 m*M* in 50 m*M* Tris–HCl buffer pH 7.5 containing 150 m*M* NaCl. The protein was concentrated and subjected to size-exclusion chromatography using a 26/60 Superdex200 High Load (HL) gel-filtration column (GE Healthcare) equilibrated with 20 m*M* HEPES pH 7.5, 150 m*M* NaCl, 1 m*M* DTT. The elution profile revealed most GlmU to be present as a trimer.

### 2.2. Crystallization, data collection and processing

GlmU crystals in the ligand-free state were grown in VDX plates (Hampton Research) by the hanging-drop vapour-diffusion method at 277 K against 1 ml reservoir solution consisting of 25 m*M* MES pH 6.5, 1.8 *M* ammonium sulfate, 5% glycerol, 5 m*M* MgCl_2_. The drops contained 2 µl concentrated protein solution (∼15 mg ml^−1^ in 20 m*M* HEPES pH 7.5, 150 m*M* NaCl and 1 m*M* DTT) and 2 µl reservoir solution. Crystals appeared in 2–3 d and grew to a size suitable for diffraction experiments in 5–7 d. Crystals were cryoprotected in 2.0 *M* ammonium sulfate, 25 m*M* MES pH 6.5 containing 25% ethylene glycol and flash-frozen in liquid nitrogen.

X-ray diffraction data were collected from these crystals using an in-house Rigaku MicroMax007HF X-ray source with a copper rotating-anode generator equipped with Varimax optics, a MAR345dtb image-plate detector and an Oxford Cryosystem 700 series cryostream. A complete data set was collected to a resolution of 3.4 Å. The data were indexed, integrated and scaled using the *XDS* program package (Kabsch, 1993 ▶). The GlmU crystals belonged to space group *I*432 (No. 211) and contained one molecule per asymmetric unit with a solvent content of ∼82% (Matthews, 1968 ▶).

### 2.3. Structure determination and refinement

The structure of GlmU from cubic crystals was determined by molecular replacement using *Phaser* (*CCP*4*i*; Read, 2001 ▶) with *M. tuberculosis* GlmU (PDB code 3dj4; Parikh *et al.*, 2008 ▶) as the search model. The top solution (LLG value 2694.4, *Z* score 46.6) showed a clear contrast with the next solution and was unambiguous for proceeding with phase refinement and model building. The initial model was built by several rounds of manual building in *Coot* (Emsley & Cowtan, 2004 ▶). The structure was refined with *REFMAC*5 (*CCP*4*i*) using the maximum-likelihood target function, employing rigid-body refinement followed by restrained refinement (Murshudov *et al.*, 1997 ▶). Owing to the low resolution of the data, a weight term of 0.3 was employed to provide tight restraints during refinement. The model quality was assessed using *PROCHECK* (Laskowski *et al.*, 1993 ▶). All figures were generated using *CHIMERA* (Pettersen *et al.*, 2004 ▶). The final model of GlmU, consisting of 439 amino acids, was refined to a resolution of 3.41 Å with an *R*_work_ of 28.5% and an *R*_free_ of 32.1%.

## 3. Results and discussion

**Figure 1:**
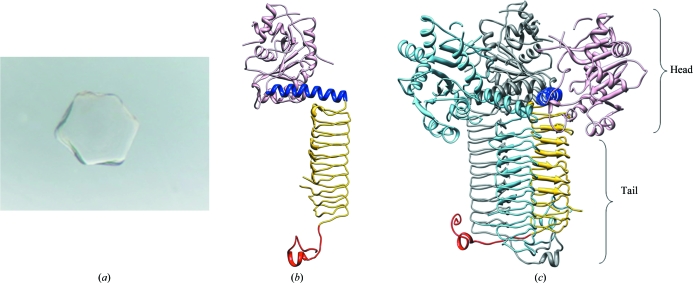
(*a*) Native crystal of GlmU obtained using ammonium sulfate as a precipitant. The crystals, which were grown in a hanging-drop setup, grew to dimensions of ∼0.3 × 0.3 × 0.3 mm. (*b*) The structure of GlmU. Each monomer contains an N-terminal domain (coloured pink) responsible for the uridyltransferase activity and a C-terminal domain with a left-handed β-helix fold (LβH; coloured gold) responsible for acetyltransferase activity. A hinge helix (coloured blue) connects the two domains and the C-terminal extensions are marked red. (*c*) GlmU forms a biological trimer. Two of the three monomers of the trimer are coloured dark grey and light blue, while the other is coloured as in (*b*). The N- and C-terminal parts of the trimer are defined as the ‘head’ and the ‘tail’.

Preliminary crystals of GlmU from *M. tuberculosis* were obtained in conditions containing ammonium sulfate as precipitant at pH 6.5. These conditions were further optimized in order to produce crystals of suitable size for diffraction experiments. The crystals grew to approximately 300 µm in all dimensions (Fig. 1 ▶ *a*). However, they consistently yielded a poor and pathological diffraction pattern at room temperature as well as when cryoprotected at 100 K. An initial data set collected to 3.8 Å resolution revealed a very high solvent content in the crystals. Hence, they were dehydrated in an attempt to improve the diffraction quality. Dehydration was carried out by soaking crystals in increasing amounts of precipitant (ammonium sulfate), sodium malonate and glycerol. This resulted in improved diffraction and a full data set could be collected to 3.4 Å resolution at 100 K. The data were processed using the program package *XDS* (Kabsch, 1993 ▶).

The crystals belonged to the cubic space group *I*432, with unit-cell parameters *a* = *b* = *c* = 285.7 Å, and contained one molecule per asymmetric unit. The structure was determined by the molecular-replacement method using *M. tuberculosis* GlmU as the search model, as detailed in §[Sec sec2]2. GlmU consists of two domains: an N-terminal uridyltransferase domain with an α/β-like fold and a C-terminal acetyltransferase domain that forms a left-handed parallel β-helix structure (LβH) with the shape of an equilateral triangular prism consisting of ten turns. The two domains are connected by a long α-­helix (Fig. 1 ▶ *b*). Although GlmU consists of 495 amino acids, a model could only be built for residues 1–472. The extended C-­terminus present in *M. tuberculosis* GlmU was not well defined in the electron-density map. The N- and C-terminal domains are known to be responsible for the uridyltransferase and acetyltransferase activities, respectively (Parikh *et al.*, 2008 ▶). The residues 149–150, 154–158, 164–177 and 200–206 that are part of the N-terminal active site are not well defined in the electron-density maps as the active site is devoid of bound ligands. Unlike the N-terminal active site, the formation of the C-terminal active site requires a trimeric arrangement (Fig. 1 ▶ *c*), as inferred from the structures of GlmU homologues (from *E. coli* and *S. pneumoniae*) bound to acetyl-CoA or CoA (Olsen & Roderick, 2001 ▶; Sulzenbacher *et al.*, 2001 ▶). The biological trimer found in the current structure of GlmU is similar to that in the GlmU homologues, except that the active-site residues 398–403 are disordered.

**Figure 2:**
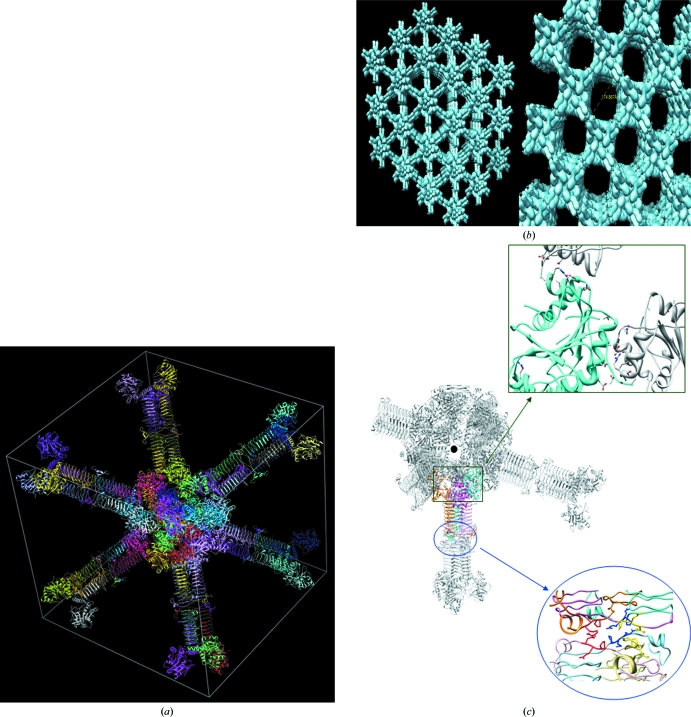
Crystal packing in the cubic form. (*a*) A view along the body diagonal of the *I*432 unit cell with unit-cell parameter 285.7 Å depicts a sparse molecular packing. (*b*) A tail-to-tail crystal contact between trimers (blue circle) along the body diagonal and a head-to-head contact between the adjacent trimers (green square) at the centre of the cube stabilizes this arrangement. (*c*) The unique arrangement of the molecules in *I*432 crystals results in large solvent channels as viewed along the diagonal (left) and the plane (right) of the cube.

**Figure 3:**
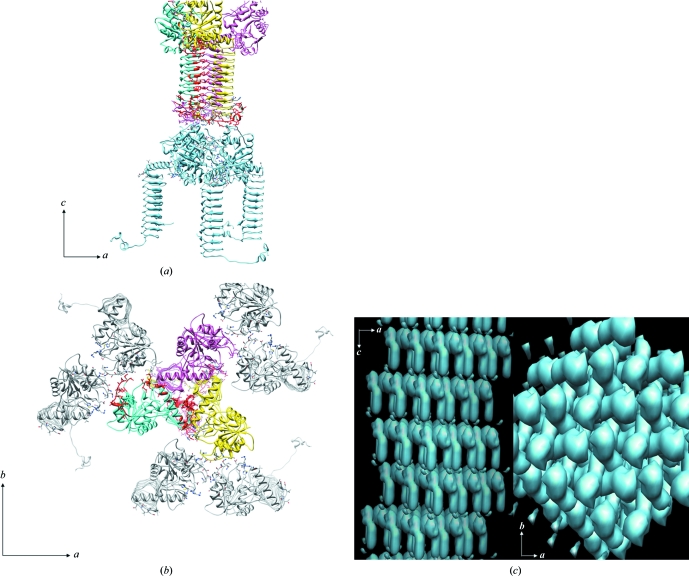
Crystal packing in the hexagonal form, generated using the coordinates of *M. tuberculosis* GlmU determined in space group *H*3 (PDB code 3dk5; unit-cell parameters *a* = *b* = 79.6, *c* = 278.0 Å). (*a*) Crystal packing showing head-to-head packing of GlmU trimers along the *c* axis. These trimers contact each other in a head-to-tail fashion. (*b*) A head-to-head arrangement of trimers results in crystal contacts in the *ab* plane. (*c*) The arrangement of molecules in the hexagonal form results in a tight packing. Crystal packing in the *ac* and *ab* planes of the crystal is depicted.

The unusually high Matthews coefficient (*V*_M_ = 9.17 Å^3^ Da^−1^) and high solvent content (>80%; Matthews, 1968 ▶) of the cubic crystals leads to the presence of large solvent channels. In contrast, most GlmU proteins crystallize in the *H*3/*H*32/*P*6_3_22 form and do not show the presence of such large channels. This led us to compare the crystal packing in the cubic and hexagonal forms. GlmU exists as a biological trimer in solution. A trimeric arrangement is common to both crystal forms. It appears that different contacts between neighbouring trimers promote crystal formation, leading to either the cubic or hexagonal forms. To facilitate a comparison of crystal contacts, we define the N- and C-terminal parts of the trimer as the ‘head’ and the ‘tail’, respectively (Fig. 1 ▶ *c*). In the cubic crystals, monomers are arranged in a trimeric fashion along each of the body diagonals, *i.e.* the crystal threefolds (Fig. 2 ▶ *a*). Two such trimers pack against each other ‘tail to tail’ (indicated by a circle in Fig. 2 ▶ *b*) and their heads face the corner and the centre of the cube. Each diagonal therefore contains four such trimers. At the centre of the cube, where the four diagonals meet, several adjacent trimers meet head to head (Figs. 2 ▶ *a* and 2 ▶ *b*) which take part in crystal contacts (indicated by a square in Fig. 2 ▶ *b*). This arrangement of molecules in the crystal results in the unusually large solvent channels in the cubic form (Fig. 2 ▶ *c*). In the hexagonal form the trimer is along the *c* axis, which is the crystal threefold, but neighbouring trimers along this axis contact in a head-to-tail fashion (Fig. 3 ▶ *a*). In addition, along the *a* and *b* axis, head-to-head contacts stabilize the packing (Fig. 3 ▶ *b*). The region involved in head-to-head contacts in the cubic form, however, is distinct from those in the hexagonal form. The compact packing appears to be a result of the head-to-tail arrangement of trimers in the *H*3/*H*32 forms. Therefore, it appears that the tail-to-tail arrangement of trimers in the cubic form results in a sparse packing in the cubic crystal form of GlmU.

*Note added in proof:* During the production of this manuscript another publication appeared on the structure of GlmU from *Mycobacterium tuberculosis* (Zhang *et al.*, 2009 ▶).

## Supplementary Material

PDB reference: GlmU, 3foq, r3foqsf

## Figures and Tables

**Table 1:** X-ray data-collection and refinement statistics Values in parentheses are for the outer shell.

Crystallization conditions	25 m*M* MES pH 6.5, 1.8 *M* ammonium sulfate, 5% glycerol, 5 m*M* MgCl_2_
Space group [No.]	*I*432 [No. 211]
Unit-cell parameter (Å)	*a* = *b* = *c* = 285.7
Data-collection statistics	
Wavelength (Å)	1.54179
Resolution (Å)	50.0–3.4 (3.50–3.41)
No. of observed reflections	230992
No. of unique reflections	25629
Completeness (%)	94.6 (91.1)
*I*/σ(*I*), overall	14.56 (4.53)
*R*_meas_† (%)	19.9 (56.4)
*R*_mrgd-*F*_‡ (%)	10.4 (22.9)
Refinement statistics	
Resolution range (Å)	48.97–3.41
No. of reflections	25629
*R*_work_§ (%)	28.5
*R*_free_¶ (%)	32.1
R.m.s.d. bonds (Å)	0.028
R.m.s.d. angles (°)	1.93
Mean *B* value (Å^2^)	49.3
No. of protein atoms	3186
No. of ions	4
Ramachandran plot: main-chain torsion-angle statistics (%)	
Most favoured	89.0
Additionally allowed	9.7
Generously allowed	0.8
Disallowed	0.6

† *R*_meas_ = 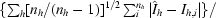 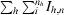, where 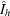 = 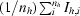.

‡ *R*_mrgd-*F*_ = 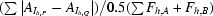, where *I*_*h,P*_ and *I*_*h,Q*_ are a measure of the quality of the reduced amplitude.

§ *R*_work_ = 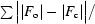 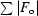, where *F*_o_ and *F*_c_ are observed and calculated structure factors, respectively.

¶ *R*_free_ was calculated using 5% of data excluded from refinement.

## References

[bb1] Barreteau, H., Kovac, A., Boniface, A., Sova, M., Gobec, S. & Blanot, D. (2008). *FEMS Microbiol. Rev.* **32**, 168–207. 10.1111/j.1574-6976.2008.00104.x 18266853

[bb2] Emsley, P. & Cowtan, K. (2004). *Acta Cryst.* D**60**, 2126–2132. 10.1107/S0907444904019158 15572765

[bb3] Kabsch, W. (1993). *J. Appl. Cryst.* **26**, 795–800.

[bb4] Laskowski, R. A., MacArthur, M. W., Moss, D. S. & Thornton, J. M. (1993). *J. Appl. Cryst.* **26**, 283–291.

[bb5] Matthews, B. W. (1968). *J. Mol. Biol.* **33**, 491–497. 10.1016/0022-2836(68)90205-2 5700707

[bb6] Mochalkin, I., Lightle, S., Narasimhan, L., Bornemeier, D., Melnick, M., Vanderroest, S. & McDowell, L. (2008). *Protein Sci.* **17**, 577–582. 10.1110/ps.073271408 PMC2248321 18218712

[bb7] Murshudov, G. N., Vagin, A. A. & Dodson, E. J. (1997). *Acta Cryst.* D**53**, 240–255. 10.1107/S0907444996012255 15299926

[bb8] Olsen, L. R. & Roderick, S. L. (2001). *Biochemistry*, **40**, 1913–1921. 10.1021/bi002503n 11329257

[bb9] Parikh, A., Verma, S. K., Khan, S., Prakash, B. & Nandicoori, V. K. (2008). *J. Mol. Biol.* **386**, 451–464. 10.1016/j.jmb.2008.12.031 19121323

[bb10] Pettersen, E. F., Goddard, T. D., Huang, C. C., Couch, G. S., Greenblatt, D. M., Meng, E. C. & Ferrin, T. E. (2004). *J. Comput. Chem.* **25**, 1605–1612. 10.1002/jcc.20084 15264254

[bb11] Read, R. J. (2001). *Acta Cryst.* D**57**, 1373–1382. 10.1107/s0907444901012471 11567148

[bb12] Sulzenbacher, G., Gal, L., Peneff, C., Fassy, F. & Bourne, Y. (2001). *J. Biol. Chem.* **276**, 11844–11851. 10.1074/jbc.M011225200 11118459

[bb14] Zhang, Z., Bulloch, E. M. M., Bunker, R. D., Baker, E. N. & Squire, C. J. (2009). *Acta Cryst.* D**65**, 275–283. 10.1107/S0907444909001036 PMC2651758 19237750

[bb13] Zhang, W., Jones, V. C., Scherman, M. S., Mahapatra, S., Crick, D., Bhamidi, S., Xin, Y., McNeil, M. R. & Ma, Y. (2008). *Int. J. Biochem. Cell Biol.* **40**, 2560–2571. 10.1016/j.biocel.2008.05.003 PMC2602953 18573680

